# GlottisNetV2: Temporal Glottal Midline Detection Using Deep Convolutional Neural Networks

**DOI:** 10.1109/JTEHM.2023.3237859

**Published:** 2023-01-19

**Authors:** Elina Kruse, Michael Döllinger, Anne Schützenberger, Andreas M. Kist

**Affiliations:** Department Artificial Intelligence in Biomedical EngineeringFriedrich-Alexander-University Erlangen–Nürnberg (FAU) 91052 Erlangen Germany; Division of Phoniatrics and Pediatric AudiologyDepartment of Otorhinolaryngology, Head and Neck SurgeryUniversity Hospital Erlangen, Friedrich-Alexander-University Erlangen–Nürnberg (FAU) 91054 Erlangen Germany

**Keywords:** Laryngeal endoscopy, glottis, deep neural networks, deep learning, midline, biomedical imaging

## Abstract

High-speed videoendoscopy is a major tool for quantitative laryngology. Glottis segmentation and glottal midline detection are crucial for computing vocal fold-specific, quantitative parameters. However, fully automated solutions show limited clinical applicability. Especially unbiased glottal midline detection remains a challenging problem. We developed a multitask deep neural network for glottis segmentation and glottal midline detection. We used techniques from pose estimation to estimate the anterior and posterior points in endoscopy images. Neural networks were set up in TensorFlow/Keras and trained and evaluated with the BAGLS dataset. We found that a dual decoder deep neural network termed GlottisNetV2 outperforms the previously proposed GlottisNet in terms of MAPE on the test dataset (1.85% to 6.3%) while converging faster. Using various hyperparameter tunings, we allow fast and directed training. Using temporal variant data on an additional data set designed for this task, we can improve the median prediction accuracy from 2.1% to 1.76% when using 12 consecutive frames and additional temporal filtering. We found that temporal glottal midline detection using a dual decoder architecture together with keypoint estimation allows accurate midline prediction. We show that our proposed architecture allows stable and reliable glottal midline predictions ready for clinical use and analysis of symmetry measures.

## I. Introduction

Vocal fold oscillations are the source for phonation [1]. Organic and functional laryngeal disorders may impact the proper oscillation function of the vocal folds, with severe costs for the healthcare system [2]. Previous research has shown that the assessment of individual vocal folds is important, as in many cases their symmetrical coordination is impaired [3], [4], [5], [6], [7], [8].

**FIGURE 1.**
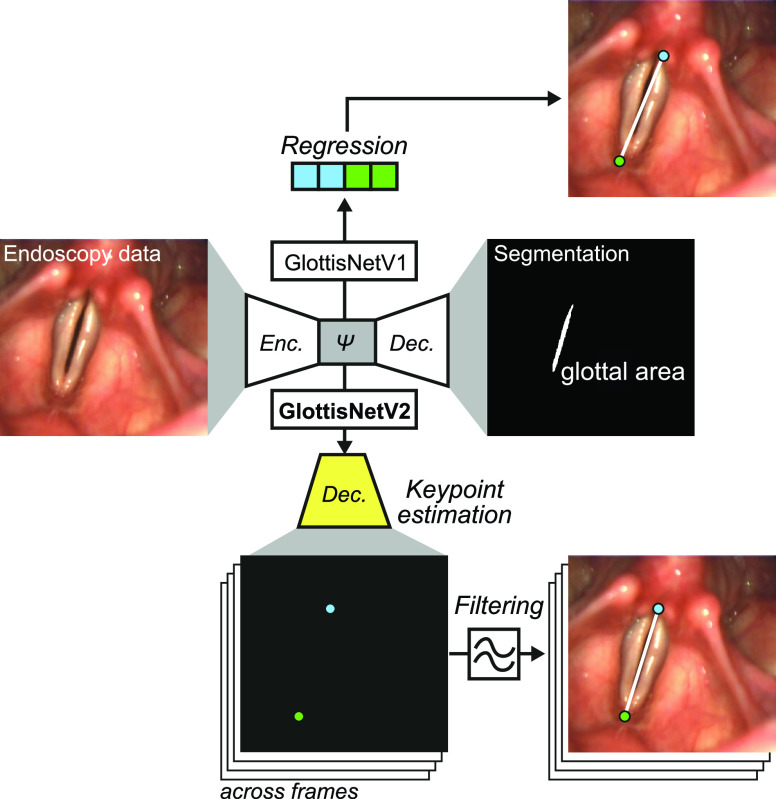
GlottisNetV2 features a novel architectural design to predict the anterior and posterior points for segmentation-agnostic midline detection. GlottisNetV1 used latent space $\Psi $-based regression resulting in inaccurate midline estimations. GlottisNetV2 features a dedicated decoder and incorporates temporal variant data and a filtering step to allow a fully automatic, highly accurate midline detection.

A major tool to quantify vocal fold oscillation patterns is high-speed videoendoscopy [9], [10]. By acquiring typically at least 4,000 frames per second, one can precisely assess the vocal fold motion in single oscillation cycles [11], [12]. An example endoscopic image is shown in Fig. 1. This footage can be utilized to quantify the vocal fold motion by segmenting the glottal area, the opening between the vocal folds [13], [14], [15]. Recently, deep neural networks have been successfully employed for glottis segmentation [13], [16] and optimized for clinical use [17].

The detection of the glottal midline is essential to analyze the symmetry of vocal fold oscillation. Commonly, the glottal midline is determined based on the glottal area: studies used the top-most and bottom-most points of the segmented glottal area [7], linear regression [18] and principal component analysis [3] to define the glottal midline. In [19], it was demonstrated that the detection of the glottal midline can be approached with a variety of computer vision methods like orthogonal distance regression, principal component analysis, image moments, and ellipse fitting. The authors further found that approaches relying on deep convolutional neural networks (MidlineNet, MobileNetV2, U-Net encoder, EfficientNetB0, and the Xception architecture) can outperform the previously listed computer vision methods [19], showing the potential of contemporary deep learning methods compared to classical computer vision models. Especially in an uncertain clinical environment with high variance, deep learning models have been shown to be superior [20], [21].

With GlottisNet (in this study referred to as GlottisNetV1), a multitask architecture was introduced that detects the two important features, glottal area and glottal midline, fully automatically and simultaneously [19]. The purpose of the multitask architecture was to gain a reliable, one-step, and end-to-end solution, that eases the workflow and lowers the number of computations. Previous works have shown that both tasks, glottal midline detection and glottis segmentation, benefit from deep neural networks [13], [19]. GlottisNetV1 is based on a U-Net-like architecture [22] containing an encoder, a latent space $\Psi $ and a decoder (Fig. 1). GlottisNetV1 uses the latent space $\Psi $ to regress the anterior and posterior points, the two anchor points that define the glottal midline, directly from the endoscopic image. With this approach it is possible to approximate the location of the glottal midline, however, it is rather imprecise on unseen data [19].

In this study, we developed GlottisNetV2, a significant enhancement of GlottisNetV1 that uses keypoint estimation using localization prediction maps instead of regression to predict the glottal midline. By utilizing a second decoder, we were able to largely increase the midline detection accuracy, while keeping the segmentation quality at baseline level. We incorporated not only spatial but also temporal variant data, and together with temporal filtering, we were able to gain highly precise and stable glottal midline predictions.

## II. Methodology

### A. Data

In this study, we utilized the benchmark dataset for automatic glottis segmentation (BAGLS) [13]. We use for the 2D experiments the full BAGLS dataset of single images (55,750 images for training and 3,500 images for testing) and for 3D experiments 30 frame-long snippets of the original underlying 640 videos. We used the anterior and posterior point annotations for the 2D images, annotated with a Python-based tool for 2D images as described previously in [19]. For the video snippets, we used a custom annotation tool written in Python, which was previously used in [19]. The anterior and posterior points were annotated as such, that the resulting glottal midline was most centered for all frames of the considered video. An example of the user interface of the annotation tool and the resulting frames is shown in Supplementary Fig. S9. Videos with non-constant positions of anterior and posterior points for all frames were removed. The final dataset for the 3D experiments consists of 532 videos for training and 41 videos for testing. Data augmentations were employed to increase the available data pool by a factor of 10. In the final model, we applied various data augmentations (rotation, horizontal flipping, blurring, random gamma, and adding Gaussian noise) using the albumentations package [23]. GlottisNetV1 and all previous versions of GlottisNetV2 only used a horizontal flip and a rotation. The 3D versions of GlottisNetV2 were all trained using all previously mentioned augmentations. For both, the 2D- and 3D experiments 10% of the training data was used for validation after each epoch. For the training of the different GlottisNetV2 versions, input images were converted to grayscale and resized. In the 2D experiments, the images had a final size of $512\times 256$ pixels. As the 3D models consist of more parameters, we resized all images to $256\times 128$ pixels. The images were normalized to a range of −1 and 1 and the glottal area segmentation masks to 0 (background) and 1 (glottis).

### B. Deep Neural Networks

**FIGURE 2.**
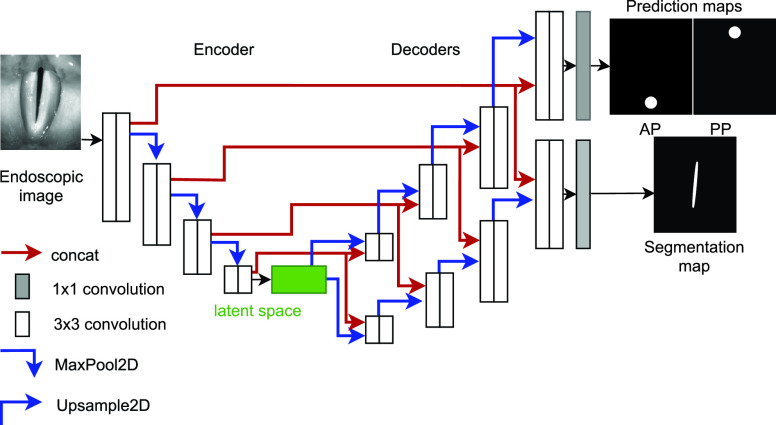
GlottisNetV2 architecture contains two decoders, one for glottal area segmentation and one for anterior (AP) and posterior point (PP) prediction using prediction maps, with a common origin in the latent space $\Psi $.

Deep convolutional neural networks were set up in TensorFlow/Keras (v. 2.5). GlottisNetV1 was set up as described previously [19]. The anterior and posterior points were regressed from the latent space by using global average pooling and a dense layer. In contrast, GlottisNetV2 uses a pixel-wise classification. The outputs were two localization prediction maps at the end of the decoder, one for the anterior and posterior points, respectively. The localization prediction map size was equal to the input image size. The anterior and posterior points were highlighted as circular regions of high-intensity values with specific radii in the prediction maps (see Fig. 2). This approach was inspired by the following studies that showed promising results: DeepLabCut [24] and SLEAP [25] utilize multiple probability maps to locate different body parts of animals and showed that landmark positioning is possible using encoder-decoder neural network architectures.

GlottisNetV1 and GlottisNetV2 use four layers in the U-Net architecture [19], [22]. The learning rate for the training of GlottisNetV1 was set to $0.2 \cdot 10^{-4}$, 64 filters and batch normalization were used. The number of filters in the training of the GlottisNetV2 versions varied between 64, 32, 16, and 8. As normalization techniques batch normalization, instance normalization, and filter response normalization were tested. Further, different learning rates were evaluated: a constant learning rate, two learning rate schedulers, and an exponentially decaying learning rate. All networks were trained using the Adam optimizer [26]. For anterior and posterior points, the MSE loss and for glottal area segmentation, the respective Dice loss [27] was used for 2D as provided by [28] and 3D as provided by [29]. As a final model, the architecture GlottisNetV2e was used. It was trained using 16 filters, instance normalization, a radius of 15 pixels, learning rate scheduler 2, and all previously mentioned data augmentation strategies.

The 2D-versions of GlottisNetV2 (GlottisNetV2a- GlottisNetV2d) are shown in Supplementary Fig. S1. GlottisNetV2e is depicted in Fig. 2. Starting from GlottisNetV2a the two problems of anterior- and posterior point prediction and the prediction of the glottal area are considered more independently from each other and each network introduces more layers that divide the two problems. In GlottisNetV2a the two prediction maps were extracted at the end of the decoder. Only the last $1\times 1$ convolution layer is different for the problem of segmentation of the glottal area and the prediction of anterior and posterior points. GlottisNetV2b divides the two problems before the second convolutional layer in the last decoding step. GlottisNetV2c has three outputs, one for the segmentation map and one each for the prediction maps of anterior and posterior points. In GlottisNetV2d the last step of the decoder is done separately for the problem of segmentation of the glottal area and the anterior and posterior point prediction. GlottisNetV2e introduces an extra decoder for the anterior point prediction, which originates in the latent space.

The architecture of GlottisNetV2 was changed for the application of three-dimensional data. Three different architectures were tested. The first architecture *GlottisNetV2 Channels* replaces the RGB data in the channel dimension of the input by multiple frames of the video. Another variant *GlottisNetV2 3DConv* uses 3D convolutions instead of 2D convolutions to include the time dimension. The third architecture *GlottisNetV2 LSTM* uses recurrent neural network layers and considers single frames subsequently. For this 2D-convolutions in GlottisNetV2 were replaced by ConvLSTM2D layers [30]. ConvLSTM2D layers were designed for the application on images and therefore use convolutions instead of matrix multiplications [30]. To avoid redundancies, memory limitations and long training times only one ConvLSTM2D layer was inserted for the previous subsequent two convolutions in each layer of the U-Net architecture (see Supplementary Fig. S8). In the last layer of the decoder, the two subsequent 2D convolutions were replaced by 3D convolutions due to memory problems. For the first comparison, all three network configurations were trained with six frames. The original network of GlottisNetV2 was also trained on the 3D data set as a reference.

The architecture of the original GlottisNetV1 was trained for 100 epochs to reach stable convergence. GlottisNetV2 in its 2D variants was trained for 30 epochs due to superior convergence behavior. The 3D-variants of GlottisNetV2 were trained for 100 epochs due to the lower amount of training data.

### C. Temporal Filtering

Temporal filtering was used to reduce the localization noise of the anterior and posterior points across frames in the videos. In particular, we utilized the moving median filter. The filter window was set to values between 6 and 18 pixels, the resulting value is the median value of the considered frames. As the number of frames in one video shall be maintained, the videos were padded before filtering. For this, the first and last frames were appended to the beginning and the end of the video multiple times.

### D. Evaluation

For evaluating the posterior and anterior points, we assessed the mean absolute percentage error (MAPE).

\begin{equation*} \text {MAPE} = \frac {100\%}{n}\sum _{i=1}^{n} \Biggl |{\frac {Y_{i}-Y_{true}}{Y_{true}}\Biggl |} \tag{1}\end{equation*}

For glottis segmentation, we used the intersection over union (IoU) metric [31].

\begin{equation*} \text {IoU}(A,B)=\frac {\text {Intersection}}{\text {Union}}=\frac {A \cap B}{A \cup B} \tag{2}\end{equation*}

To evaluate the temporal filtering method for the position of the anterior and posterior points, we calculated the standard deviation of the Euclidean distance between the predicted point and the real position.

### E. Code Availability

All relevant code for training and evaluating the GlottisNetV2 variants presented in this study are openly available at https://github.com/ankilab/GlottisNetV2. The anterior and posterior point annotations for the video snippets are available on https://zenodo.org/record/6938457.

## III. Results

### A Two-Decoder Architecture Together With Prediction Map Keypoint Estimation Outperforms State-of-the-Art Glottal Midline Detection

A.

**FIGURE 3.**
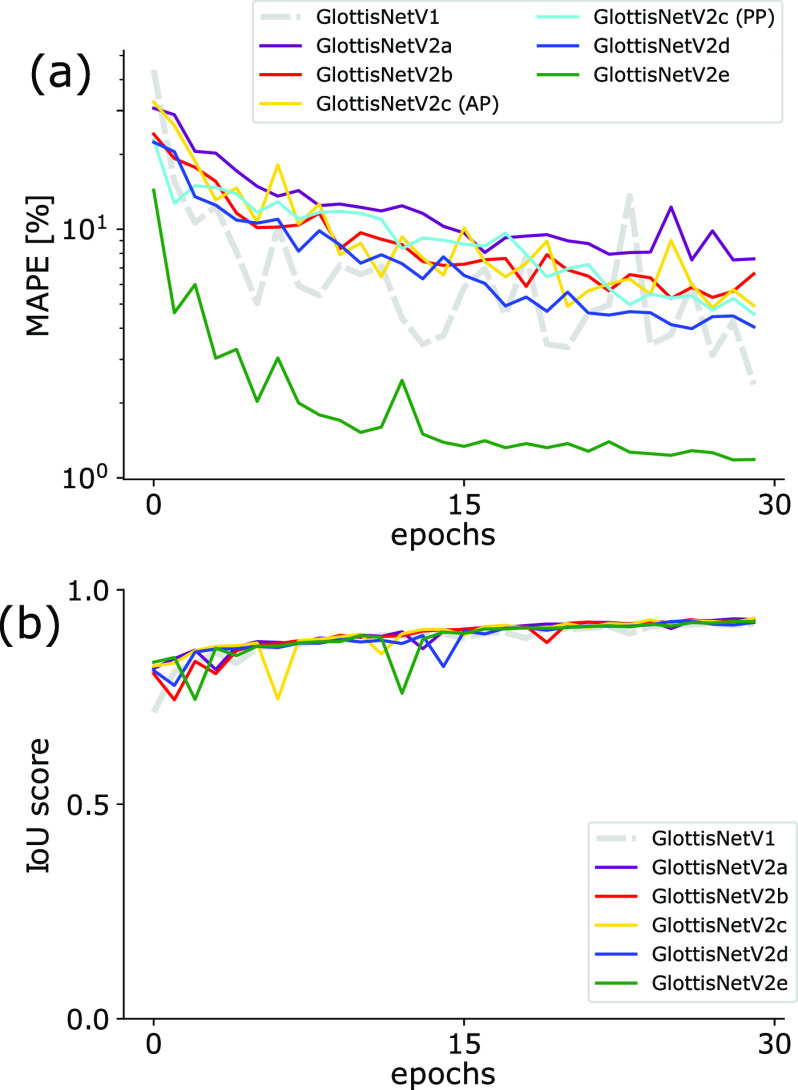
GlottisNetV2e outperforms other architectural designs. (a) Epochs vs. validation performance for anterior/posterior point prediction (MAPE) across architecture variants. (b) Epochs vs. validation performance for glottal area segmentation (IoU) across architecture variants.

The glottal midline is defined by the anterior and posterior key points in the endoscopic image (Fig. 1), allowing a glottis segmentation unbiased midline detection, which is based on anatomical constraints. In contrast to GlottisNetV1, where the two key points were estimated using regression (Fig. 1 top), we evaluated if the two key points can be estimated using pixel-wise classification, similar to pose estimation [24], [25], [32]. We evaluated a library of different fully convolutional architectures (Supplementary Fig. S1 and Fig. 2). In this case, we measured the performance of each architecture in keypoint detection and glottis segmentation using the MAPE and the IoU score on the validation data set, respectively. We found that all architectures show similar performance compared to GlottisNetV1, but when using two separate decoders with the same origin in the latent space (Fig. 2 and (referred to as GlottisNetV2e)), the new methodological approach together with the proposed architecture largely outperforms GlottisNetV1 in keypoint detection (Fig. 3a), while performing on par in terms of glottis segmentation (Fig. 3b).

**FIGURE 4.**
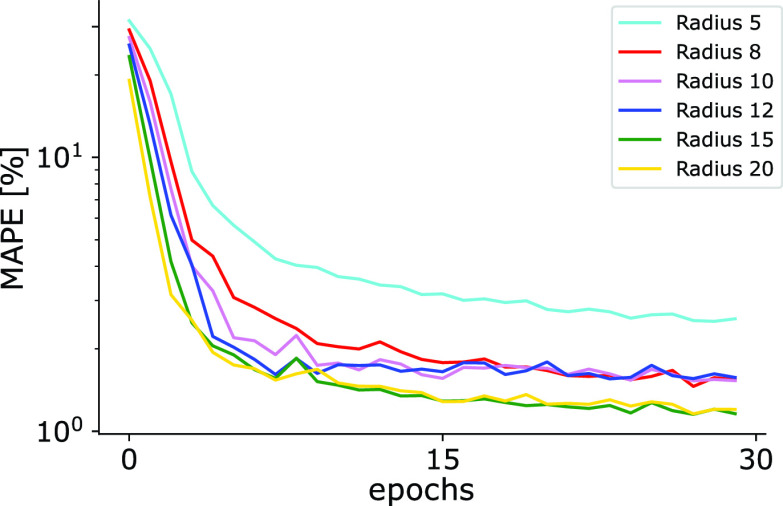
Training behavior of GlottisNetV2, when trained with different radii for the anterior and posterior point location in the target probability maps. We show training epochs vs. validation performance for anterior/posterior point prediction by measuring the MAPE.

For faster training and better usability of the neural network, the number of base layer filters was reduced from originally 64 to 16, which showed similar convergence behavior (see Supplementary Fig. S2). Further, we found out that the size of the radii in the prediction maps for anterior and posterior points influences the performance (see Fig. 4).

Small radii lead to insecurities in the localization of anterior and posterior points (radius 5: MAPE of 2.55% on the validation data set). Intermediate radii show slight improvements in the prediction of the anterior and posterior points (radius of 8, 10, and 12, MAPE: ca. 1.4%), and large radii show even better performances (see radius 15 and 20 (MAPE of 1.15%) in Fig. 4). Between radii of 15 and 20 pixels, no further improvements are visible. As the radius in the prediction maps for the anterior and posterior point (> 20 px) was becoming rather large compared to the width of the input image (256 pixels) and the performance already sufficiently increased, we stopped evaluating at a radius of 20 pixels. Our studies were inspired by the study in [24], where body parts of insects were localized using prediction maps with regions of small radii. For further evaluations, we used a radius of 15 pixels with an image size of $512\times 256$ pixels. The resulting prediction maps for anterior and posterior points for radii of 5, 10, 15, and 20 pixels are depicted in Supplementary Fig. S4.

The originally used batch normalization was replaced by instance normalization because of the smoother and faster convergence behavior of the MAPE metric, while the segmentation quality remains stable (see Supplementary Fig. S3).

Further, a learning rate scheduler was introduced to speed up the training in the beginning and to yield a smoother convergence of the MAPE metric (see Supplementary Fig. S5).

The introduction of further and more sophisticated augmentations did not lead to large improvements (Supplementary Fig. S6).

Table 1 shows a summary of the used hyperparameters of the final model of GlottisNetV2 for comparison with GlottisNetV1.

**TABLE 1:** Final Hyperparameters Used for the Training of GlottisNetV2

	Parameter
Architecture	GlottisNetV2e
Number of filters	16
Normalization technique	Instance Normalization
Radius	15 px
Learning rate	Learning rate scheduler 2 (Suppl. Fig. 5)
Augmentations	Gaussian noise, Blur, Random Gamma, Random Brightness

**FIGURE 5.**
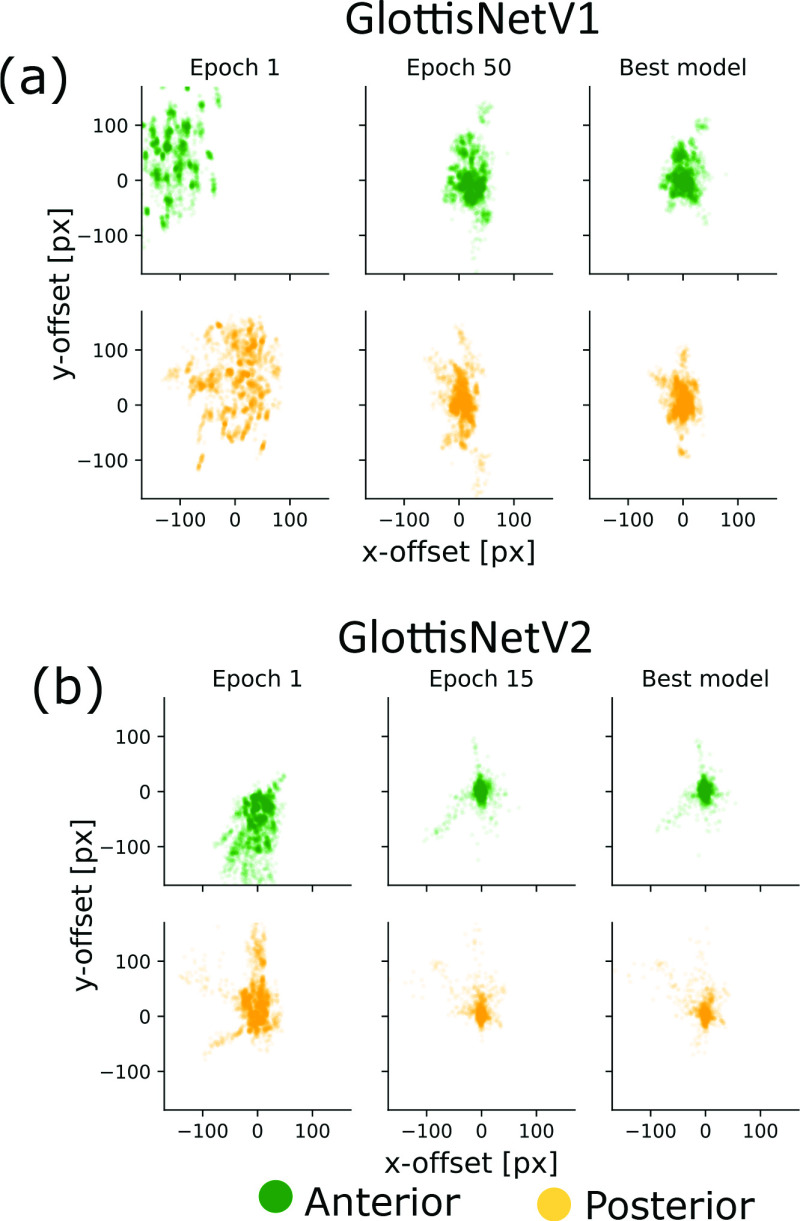
Comparison of the convergence behavior of the anterior and posterior point prediction of GlottisNetV1 and the final version of GlottisNetV2 in pixels. The distances between ground truth and the predicted location of anterior and posterior points are illustrated after the first epoch, after half of the training process is finished (50 epochs for GlottisNetV1 and 15 epochs for GlottisNetV2) and for the final model.

We found, that the MAPE of the final GlottisNetV2 converges faster and is overall more precise than GlottisNetV1 (shown in Fig. 5b by the more concentric points clouds of GlottisNetV2 in comparison to GlottisNetV1). The localization of the anterior and posterior points is more precise after 30 epochs of training of GlottisNetV2 than after 100 epochs of training of GlottisNetV1.

By comparing the MAPE scores of all images in the test data set, we found a significant improvement in the median MAPE score, the first and the third quartile, and the minimum and maximum MAPE score of GlottisNetV2 in comparison to GlottisNetV1 (see Table 2). The results of the same evaluation on the validation data set can be seen in Supplementary Fig. S7.

**TABLE 2:** Performance of the Anterior and Posterior Point Prediction of the Final Version of GlottisNetV2 Evaluated on the Test Data Set (MAPE in %)

	Min	1st quartile	Median	3rd quartile	Max
GlottisNetV2 (ours)	0.11	1.08	1.85	3.75	7.73
GlottisNetV1	0.57	3.89	6.30	11.59	23.11

### B. Time-Variant Glottal Midline Prediction

**FIGURE 6.**
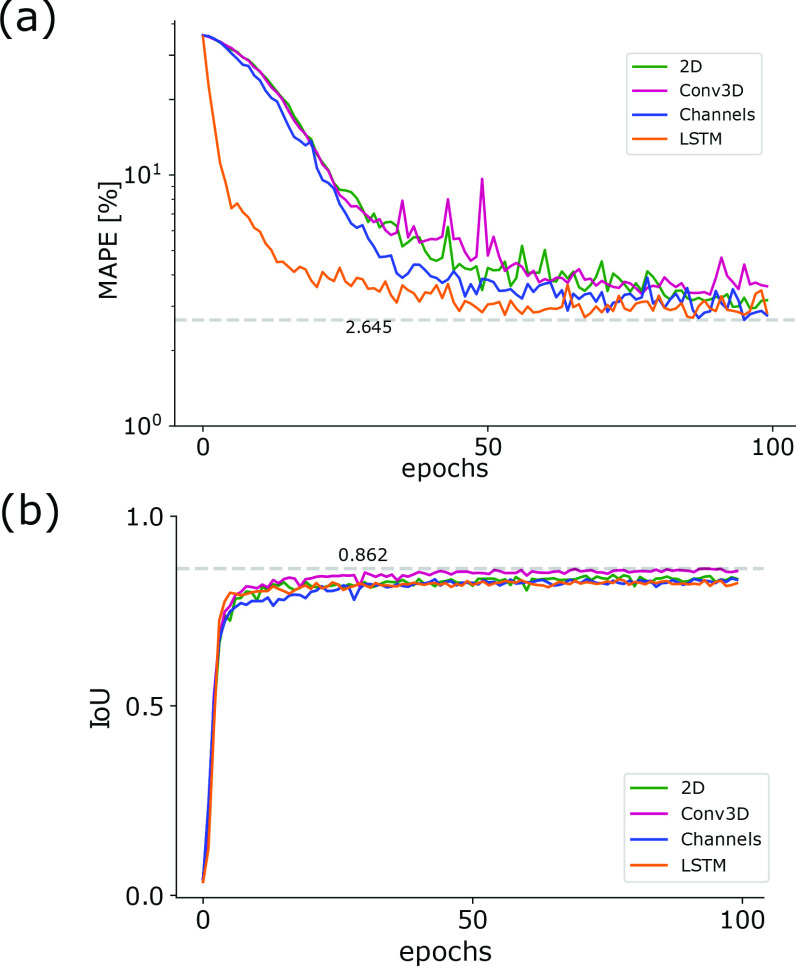
Training behavior of the 3D-versions of GlottisNetV2 (epochs vs. validation performance) for anterior and posterior point prediction (a) and glottal area segmentation (b).

We next asked if incorporating temporal data enhances the midline accuracy. As the vocal fold oscillation is a complex temporal variant, and physiological phenomenon, temporally close anatomical information improves midline detection [19]. Therefore, we investigated which architectural design is best suited to incorporate temporal information. We either used multiple channels with 2D convolutions, 3D convolutions, or LSTM-propagated 2D convolutions (see Methods and Supplementary Fig. S8). As the image size had to be decreased from a size of $512\times 256$ pixels to a size of $256\times 128$ pixels for saving memory resources (see Methods), the radius was adapted to a size of 7.5 pixels for the following evaluations to remain comparable to the 2D versions of GlottisNetV2. Moreover, we used a constant learning rate of 0.2 10^−3^ for the 3D-experiments, as the number of epochs was changed due to the smaller number of videos for training. Evaluating these three 3D-variants of GlottisNetV2, we found that the final MAPE scores are similar for all variants of GlottisNetV2 (see Fig. 6 (a)). *GlottisNetV2 LSTM* needs the fewest epochs to converge. The duration of one epoch of training nevertheless is considerably higher. *GlottsinetV2 LSTM* needs 1600 seconds for one epoch of training. In contrast, the duration of one training epoch for *GlottisNetV2 Channels* is 478 seconds and for *GlottisNetV2 3DConv* is 660 seconds. Further, we found that the segmentation shows little performance differences across the different variants of GlottisNetV2 (Fig. 6 (b)). Generally, the IoU score on the validation dataset converges to lower values with the novel 3D data set (0.93 on the BAGLS dataset vs. 0.86 on the novel 3D dataset). Similarly, the smallest MAPE score was 1.19% on the 2D BAGLS dataset vs. 2.64% on the novel 3D dataset). This is likely due to the smaller dataset size compared to the 2D BAGLS dataset.

### C. Improving Glottal Midline Stability by Increasing Temporal Context

To determine the inference noise of the glottal midline across time, we examined the mean of the standard deviation of the Euclidean distance between the prediction and the true position of anterior and posterior points (see Table 3). We found that both anterior and posterior point predicted by *GlottisNetV2 LSTM* show most movements of the glottal midline. Interestingly, the 2D-reference of GlottisNetV2 shows convincing results in the determination of the glottal midline, and also only small deviations are noticeable.

**TABLE 3:** Standard Deviations [px] of the Predicted Positions of Anterior and Posterior Points by the 3D Versions of GlottisNetV2 and a 2D Reference

	LSTM	3DConv	Channels	2D
Anterior	2.51	1.89	2.00	1.12
Posterior	2.21	0.85	1.39	1.28

**FIGURE 7.**
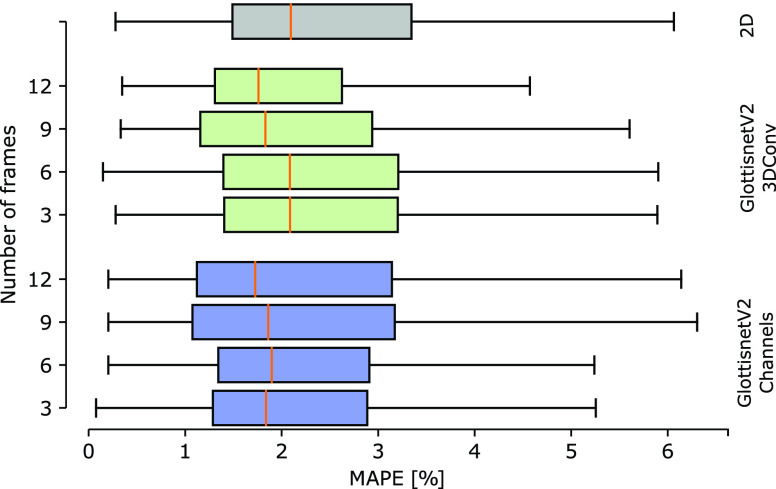
Distribution of the MAPE score on the test set across GlottisNetV2 variants (2D in gray, 3D with 2D convolutions and multiple channels in blue, and 3D with 3D convolutions in green) trained with temporal context by passing 3, 6, 9 or 12 frames to the network.

To further benefit from the use of temporal variant, i.e. 3D data, the architectures *GlottisNetV2 Channels* and *GlottisNetV2 3DConv* were trained with increased temporal context (9 and 12 frames) and as a comparison with 3 frames. To compare all architectures, we determined their MAPE score distribution on previously unseen data (see Fig. 7). *GlottisNetV2 LSTM* was not considered further because of considerably long training times and convergence against much higher values of the MAPE score. The best performance is achieved when using *GlottisNetV2 3DConv* with 12 frames (median MAPE score of 1.76%). Although the median MAPE score and the 1st quartile are similar to *GlottisNetV2 Channels*, the 3rd quartile and the highest detected MAPE score are lower, and the distribution, in general, more compact. *GlottisNetV2 Channels* trained with 9 and 12 frames also shows an improvement in the median MAPE score and the 1st quartile. The 3rd quartile, however, shows an increase. These results suggest that increased temporal context is improving the prediction accuracy, however, only in architectural designs that retain temporal context across the full feed-forward pass. The observed 12 frames are also close to one average oscillation cycle, allowing us to incorporate the full oscillation behavior and approach the true underlying oscillation center.

### D. Stabilizing the Glottal Midline Using Temporal Filtering

**FIGURE 8.**
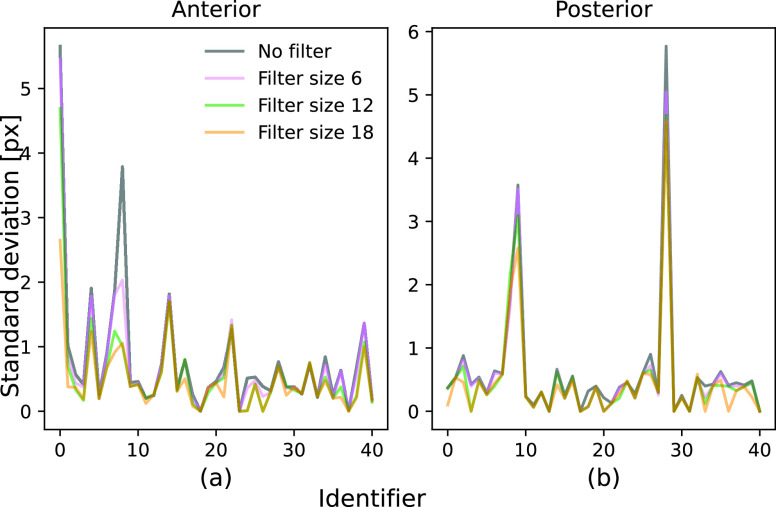
Standard deviation of the euclidean distance between ground truth and predicted location of anterior and posterior points evaluated on the test set after applying the moving median filter with varying filter windows [px].

Finally, the inference noise between adjacent frames was reduced by using temporal filtering. We evaluated the jitter in glottal midline prediction using the standard deviation of the euclidean distance in Fig. 8. For this evaluation, we used GlottisNetV2 with 3D convolutions and 12 adjacent frames, as it achieved the best performance results in the previous experiments. We show that temporal filtering using the moving median is lowering the standard deviation, and thus, the instability of the glottal midline across time. In general, the anterior point seems to be more susceptible to noise than the posterior point (Table 3). This is also maybe due to the fact, that the posterior point is easier to determine than the anterior point, as there is some variability in determining the anterior point manually in endoscopic images. Nevertheless, we found that by applying a significantly large window of 12 to 18 frames, we are able to reduce the instability over time.

## IV. Discussion

In this work, we investigated the use of localization probability maps instead of direct point regression (Fig. 1). We further divided internally the two tasks - glottal area segmentation and prediction of the anterior and posterior point - by using two separate decoders, while still relying on a single forward pass through the architecture. This led to a significant performance boost of anterior and posterior point prediction compared to the previously suggested GlottisNetV1 architecture introduced in [19]. We showed that landmark prediction as used in animal tracking or pose estimation [24], [25] is feasible for locating the anterior and posterior points in endoscopy images of the vocal folds.

Noticeably, the prediction of anterior and posterior points is more difficult than the segmentation of the glottal area, as the performance stays constant despite huge changes in the architecture. Our findings are in line with previous works that show the stability of glottal area segmentation across various architectural designs [17], [33].

Vocal fold oscillation is a temporal variant phenomenon. Therefore, it feels natural to utilize architectures that consider the temporal context. However, for both, glottal midline detection and glottis segmentation are not a necessity [13], [16], [17], [19]. Excellent work of Fehling and colleagues showed the importance of time, in either direction or bidirectional, as they found a constant gain in glottal area segmentation performance [33], while in our hands we barely see an increase in segmentation performance. This discrepancy is maybe due to a different dataset used for model training or the use of RGB images in [33], whereas we use grayscale images only. However, evidence for better performance using temporal variant data in glottal midline detection has been shown in [19]. The authors found that summing across glottal area segmentations for segmentation-dependent glottal midline detection improved the midline detection with conventional computer vision methods by 7.6%, whereas significant improvement could be shown using Conv2DLSTM layers in a custom deep neural network.

A huge advantage of GlottisNetV2 compared to the segmentation-based glottal midline determination using computer vision approaches [7], [18], [19] is the reliable determination of the glottal midline independently of the glottal area because it prevents biases towards the segmentation quality and the pathology [19]. On the other side, a drawback compared to the previously mentioned computer vision methods is the high computational complexity of the training process and the dependency on a huge labeled training data set. With GlottisNetV2, a single forward pass is necessary to determine all relevant information for phonovibrograms, a 2D representation of the vocal fold oscillation across time [7]. Using our improved and reliable architecture, we believe that this will lead to better adaptation of the unbiased phonovibrogram in further practice.

We show that GlottisNetV2 with suitable hyperparameters is a robust architecture (Fig. 2 and 5). We believe that further improvements can be made by providing more diverse 3D data, which was limited in our study to 640 videos from the BAGLS dataset [13]. Future research should also aim at the broad evaluation of GlottisNetV2 on a larger body of clinical data, to determine the failed cases important for long-term use in a clinical context.

Multitask architecture is a general concept that leverages also the inductive bias introduced by multiple, related tasks [34]. A plethora of potential multitask architectural designs have been described [35], also showing that each multitask approach needs to be tailored to the specific environment. We can show with this and our previous study [19], that a common representation in a latent space is crucial for shared knowledge in line with contemporary works [36]. We believe that this strategy can be further utilized across different medical tasks, such as multimodal data integration or multi-object detection.

## V. Conclusion

With GlottisNetV2, we present a neural network for the simultaneous prediction of the glottal midline and the segmentation of the glottal area. We determine the glottal midline by predicting the anterior and posterior part in endoscopic images using a localization probability map in contrast to earlier approaches: when replacing the previously used regression of the anterior and posterior point coordinates by a pixel-wise classification and introducing an additional decoder for this task, we improved and sped up the prediction of anterior and posterior points. Using sophisticated hyperparameter tuning, we were able to optimize the training process and the stability of the glottal midline prediction. By using 3D data and temporal filtering, the overall performance could be improved. GlottisNetV2 is a crucial step forward towards clinical applicability of not only glottal area-derived measures but also in the comprehensive analysis of the oscillation symmetry.

## Supplementary Information

We provide nine Supplementary Figures in the Supplementary Information document.

Supplementary materials
