# Supplementary material for: GlottisNetV2: Temporal Glottal Midline Detection Using Deep Convolutional Neural Networks
Source: IEEE J Transl Eng Health Med. 2023 Jan 19;11:137–44. doi: 10.1109/JTEHM.2023.3237859 (PMC9933989; doi:10.1109/JTEHM.2023.3237859)
Supplement: Supplementary materials [file supp1-3237859.pdf]

GlottisNetV2: Temporal Glottal Midline Detection using Deep Convolutional Neural Networks

E Kruse, M Döllinger, A Schützenberger, A M Kist

Supplementary Information

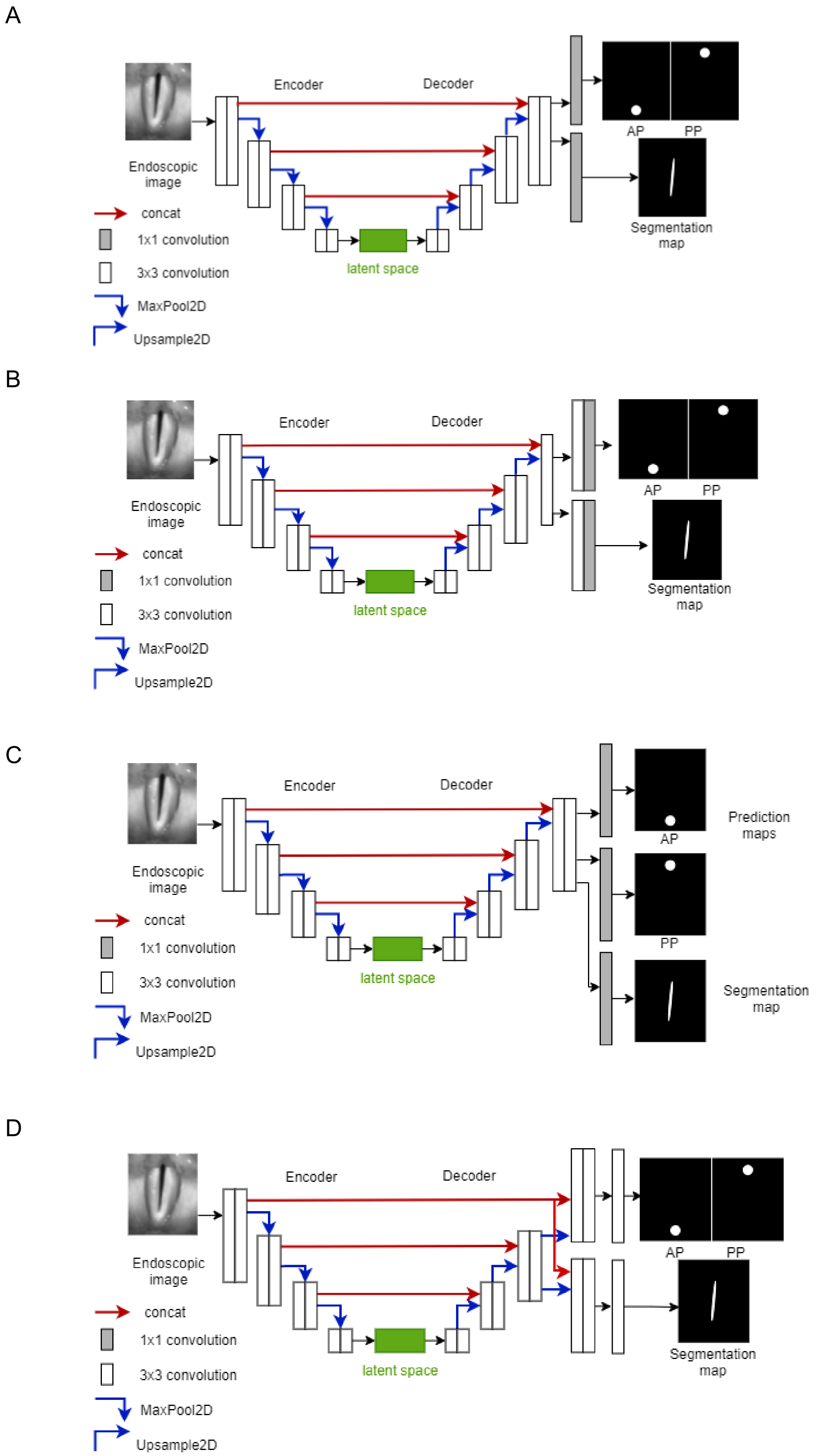

Supplementary Figure S1: Overview of evaluated GlottisNetV2 variants.
A) GlottisNetV2a, B) GlottisNetV2b, C) GlottisNetV2c, D) GlottisNetV2d

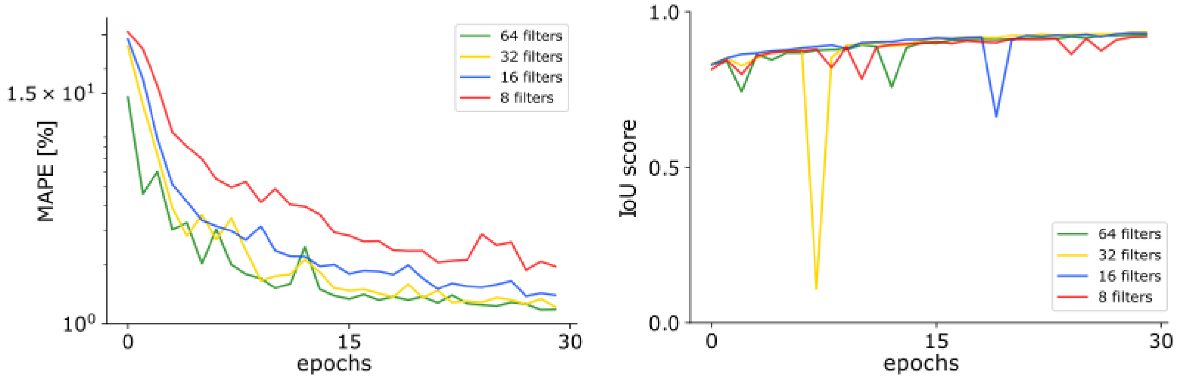

Supplementary Figure S2: Model capacity affects glottal midline prediction, but not glottal segmentation. Training behavior across different numbers of filters. MAPE and IoU evaluated on validation data set.

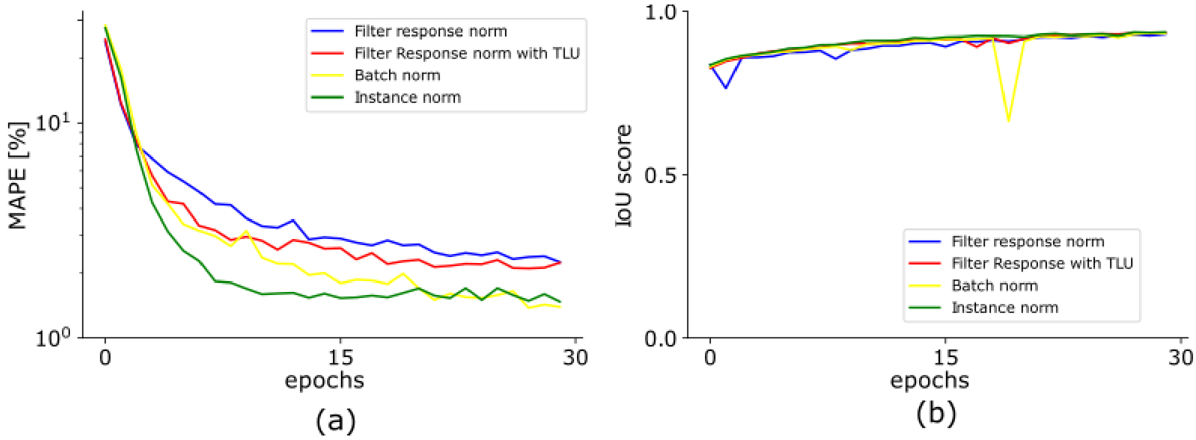

Supplementary Figure S3: Training behavior with different normalization techniques. MAPE and IoU evaluated on validation data set.

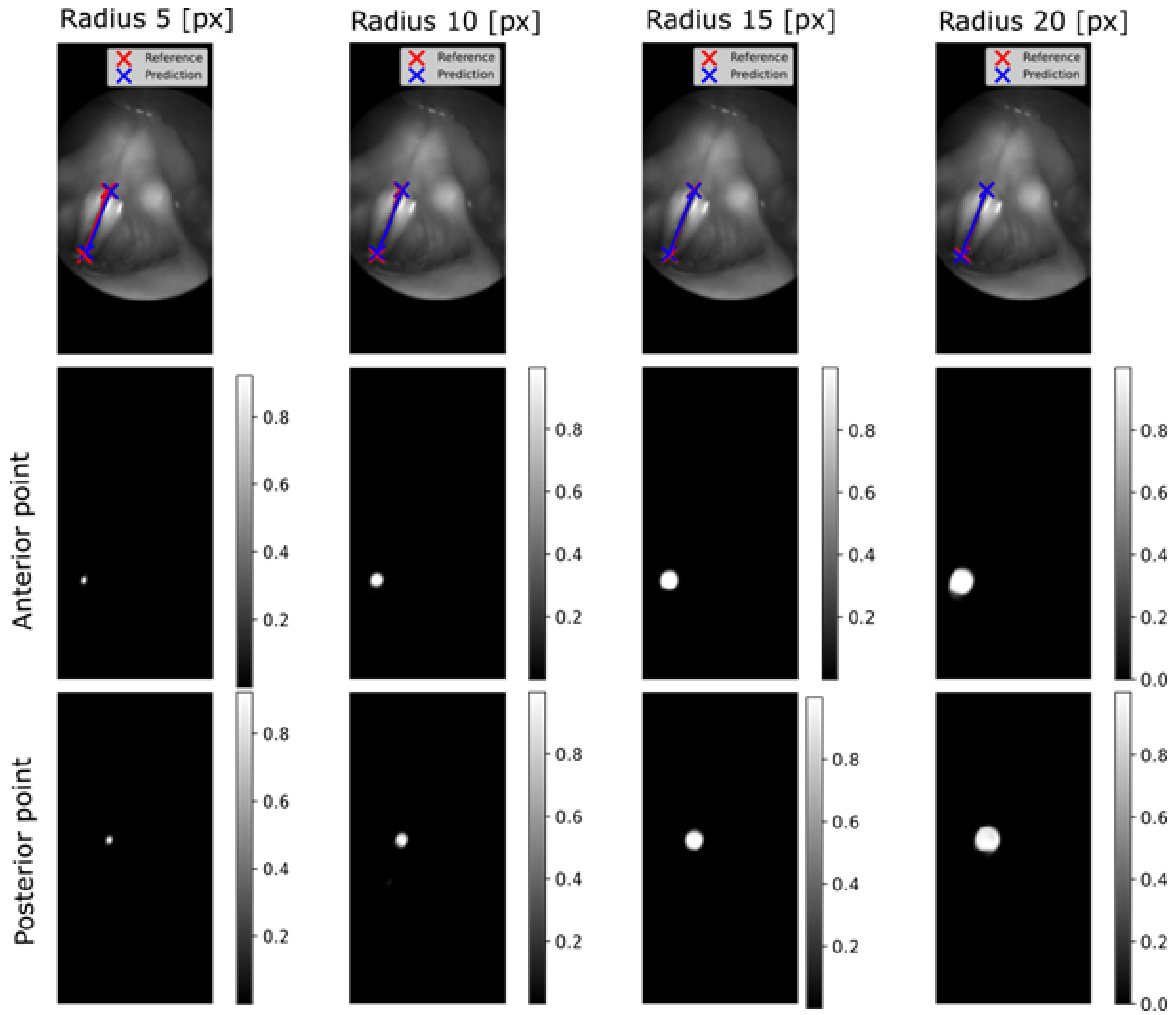

Supplementary Figure S4: Examples of the prediction maps returned by GlottisNetV2e when trained with different radii. The color depicts the probability of the anterior and posterior point being in that particular reagion.

Epoch < 5	1e-3
Epoch < 10	2e-4
Epoch < 15	0.5e-4
Epoch < 20	0.2e-4
Epoch < 25	1e-5
Epoch < 30	1e-6

Learning rate scheduler 1

Epoch < 5	1e-3
Epoch < 15	2e-4
Epoch < 25	1e-4
Epoch < 30	1e-5

Learning rate scheduler 2

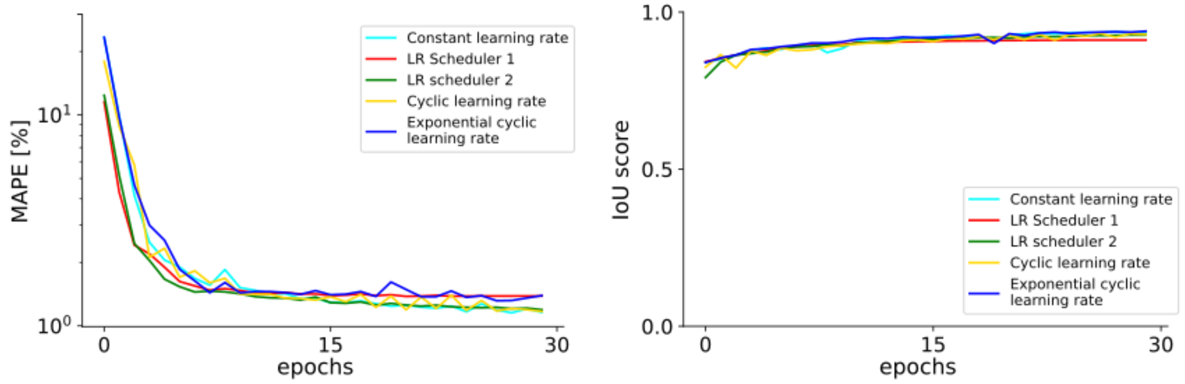

Supplementary Figure S5: Learning rate schedule affect slightly training behavior. MAPE evaluated on validation and training data set.

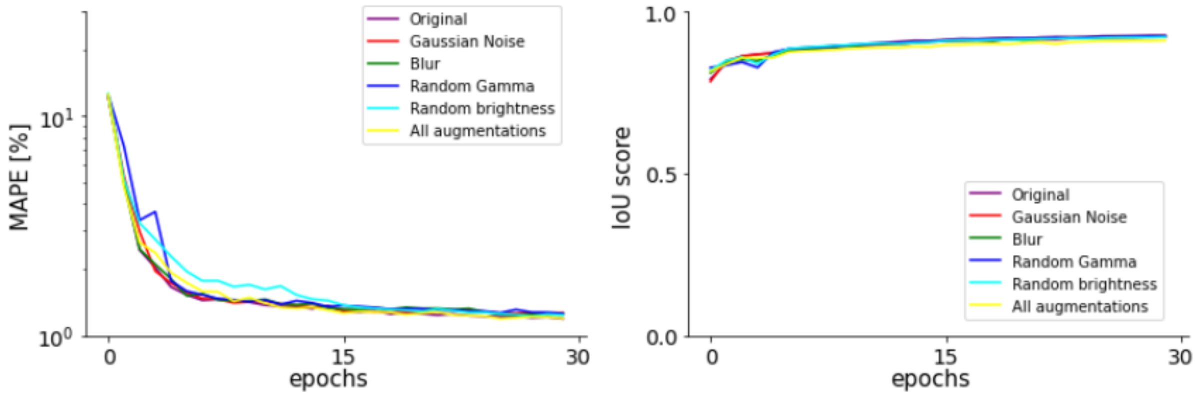

Supplementary Figure S6: Different Augmentations compared with the previously used version of GlottisNetV2, where only a horizontal flip and a rotation were used. MAPE evaluated on validation and training data set.

	Minimum	1st quartile	Median	3rd quartile	Maximum
GlottisNetV2	0.07	0.59	0.86	1.27	2.28
GlottisNetV1	0.20	1.09	1.54	2.25	3.98

Supplementary Figure S7. Comparison of the final version of GlottisNetV2e and GlottisNetV1 using the validation data set.

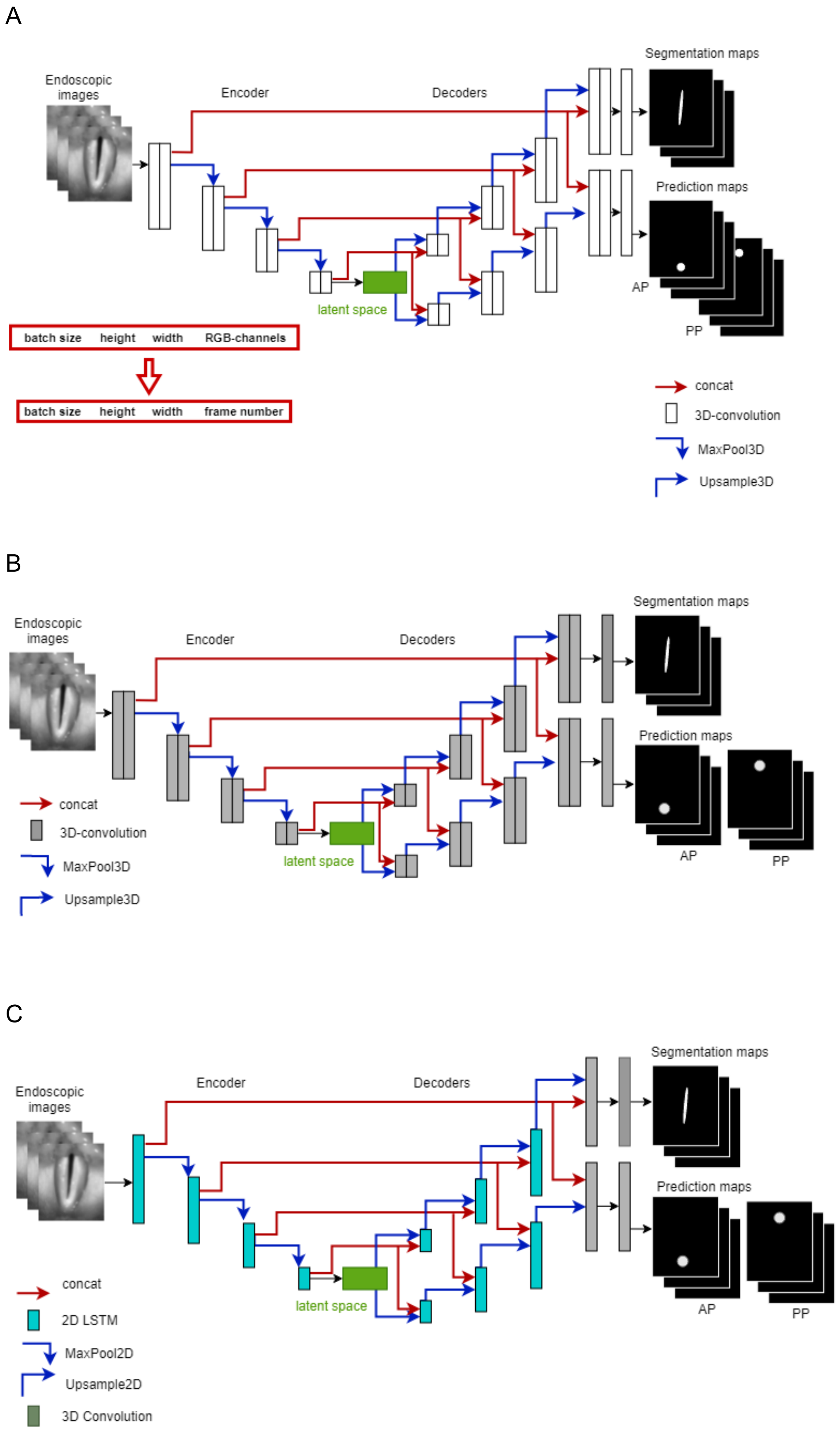

Supplementary Figure S8: Overview of evaluated GlottisNetV2 3D-variants.
A) GlottisNetV2 Channels, B) GlottisNetV2 3DConv, C) GlottisNetV2 LSTM

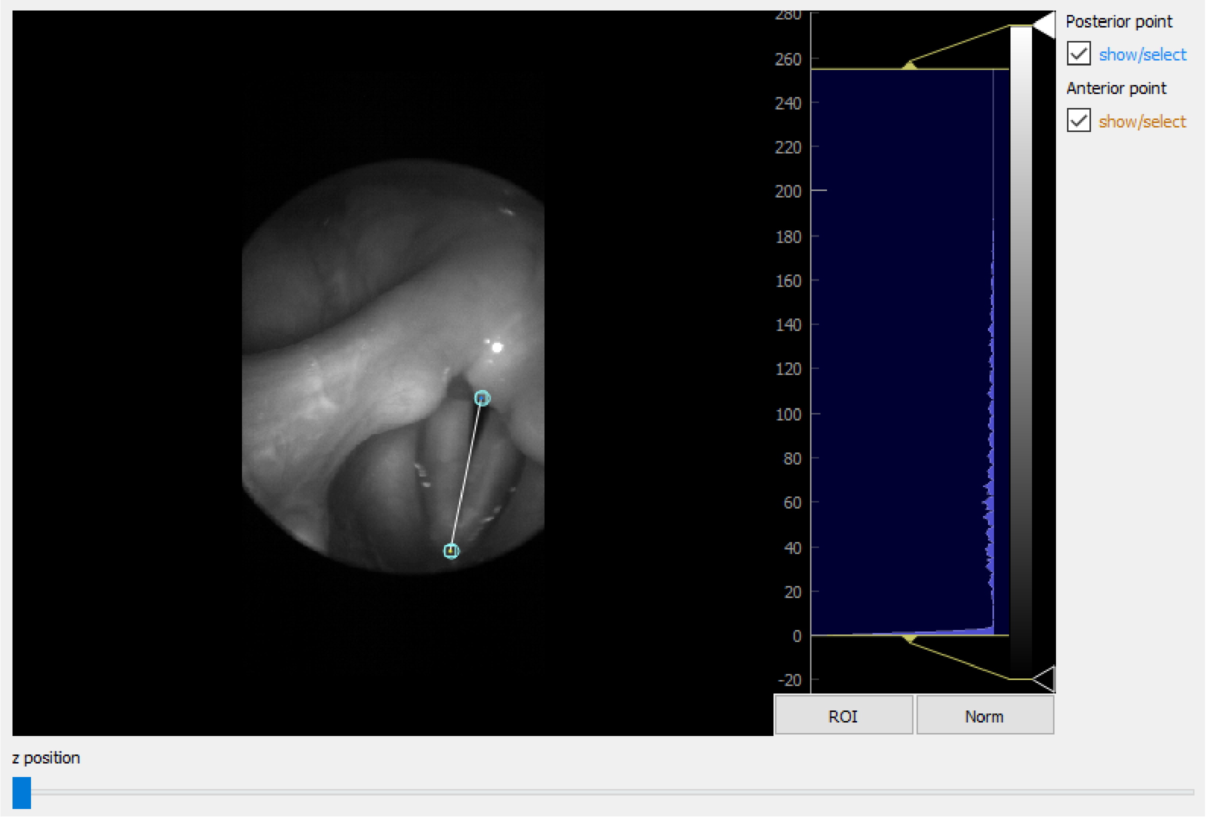

(a)

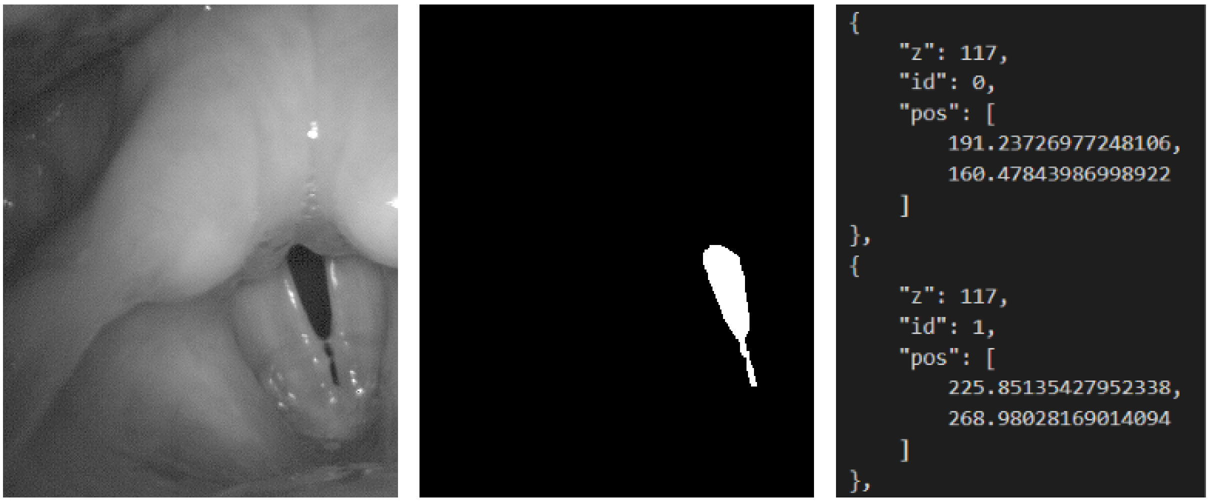

(b)

Supplementary Figure S9: User interface of the annotation tool and resulting training data for the 3D-variants of GlottisNetV2. (a) Screenshot of annotation tool. (b) Example of an input image of the glottis, the segmentation of the glottal area, and the coordinates of anterior and posterior points of one frame.
